# Probiogenomic In-Silico Analysis and Safety Assessment of *Lactiplantibacillus plantarum* DJF10 Strain Isolated from Korean Raw Milk

**DOI:** 10.3390/ijms232214494

**Published:** 2022-11-21

**Authors:** Sujatha Kandasamy, Jayeon Yoo, Jeonghee Yun, Kil-Ho Lee, Han-Byul Kang, Ji-Eun Kim, Mi-Hwa Oh, Jun-Sang Ham

**Affiliations:** Animal Products Research and Development Division, National Institute of Animal Science, Rural Development Administration, Wanju 55365, Republic of Korea

**Keywords:** *Lactobacillus*, probiotic, whole genome sequence, bacteriocin, stress-related enzymes, safety assessment, mobile genetic elements

## Abstract

The whole genome sequence of *Lactiplantibacillus plantarum* DJF10, isolated from Korean raw milk, is reported, along with its genomic analysis of probiotics and safety features. The genome consists of 29 contigs with a total length of 3,385,113 bp and a GC content of 44.3%. The average nucleotide identity and whole genome phylogenetic analysis showed the strain belongs to *Lactiplantibacillus plantarum* with 99% identity. Genome annotation using Prokka predicted a total of 3235 genes, including 3168 protein-coding sequences (CDS), 59 tRNAs, 7 rRNAs and 1 tmRNA. The functional annotation results by EggNOG and KEGG showed a high number of genes associated with genetic information and processing, transport and metabolism, suggesting the strain’s ability to adapt to several environments. Various genes conferring probiotic characteristics, including genes related to stress adaptation to the gastrointestinal tract, biosynthesis of vitamins, cell adhesion and production of bacteriocins, were identified. The CAZyme analysis detected 98 genes distributed under five CAZymes classes. In addition, several genes encoding carbohydrate transport and metabolism were identified. The genome also revealed the presence of insertion sequences, genomic islands, phage regions, CRISPR-cas regions, and the absence of virulence and toxin genes. However, the presence of hemolysin and antibiotic-resistance-related genes detected in the KEGG search needs further experimental validation to confirm the safety of the strain. The presence of two bacteriocin clusters, sactipeptide and plantaricin J, as detected by the BAGEL 4 webserver, confer the higher antimicrobial potential of DJF10. Altogether, the analyses in this study performed highlight this strain’s functional characteristics. However, further in vitro and in vivo studies are required on the safety assurance and potential application of *L. plantarum* DJF10 as a probiotic agent.

## 1. Introduction

In recent years, the increasing awareness of probiotic therapy that offers health benefits beyond nutrition led to a surge in global probiotic foods consumption. Probiotics are defined as “live microorganisms which, when ingested in adequate amounts, confer a health benefit on the host” [1]. Probiotic bacteria, particularly strains of the genera *Lactobacillus*, are usually isolated from fermented foods, gastrointestinal and vaginal tracts of humans and animals, plant materials and soil [2]. Certain lactobacilli strains are widely employed as starters in several food and beverage industries due to generally recognized as safe (GRAS) and Qualified Presumption of Safety (QPS) status besides their unique properties for such applications [3,4]. Concerning probiotic properties, certain lactobacilli strains are used as health therapies, such as immune modulation, to cure gastric diseases and prevent harmful bacterial colonization [1,5]. Such potentially probiotic strains also can survive under low pH and bile stress, have higher adhesion to the intestinal epithelial cells and produce linoleic acid and exopolysaccharides [6,7].

Among the various probiotic lactobacilli, *Lactiplantibacillus plantarum* (*L*. *plantarum*), formerly known as *Lactobacillus plantarum*, can survive in varied ecological niches, i.e., all types of fermented foods and gut microsystems of human and animals [2,8,9]. Besides safety, *L. plantarum* is easy to culture and is therefore of great interest to researchers in the food industry. *L. plantarum* is widely used as a probiotic and/or microbial starter in food and feed industries for its beneficial effects, such as antioxidant, antibacterial, cholesterol-reduction, immunomodulatory, antihypertensive, boosting epithelial defense functions, and sustaining gut microbiota homeostasis [5,7,10]. However, a few issues relating to consumer safety have been stated around the intake of probiotic LAB, especially in the immunocompromised population [11]. It should be emphasized that the function and safety of probiotics are strain-specific and cannot be generalized. Therefore, to ensure the safety of probiotic food, it is vital to strictly evaluate the safety and possible adverse interactions of the probiotics with the host, as probiotic products are generally meant for daily consumption.

The demand for consumer satisfaction with probiotic products necessitates the development of speedy and reliable techniques to evaluate the efficacy and safety of probiotic strains. In recent years, the whole-genome sequence coupled with bioinformatics analysis has gained more attention as it enables a deep understanding of the genome, diversity and evolution of probiotic candidates with precise identification and safety evaluation [12]. The safety, adaptation and probiotic strain properties (cell membrane composition, epithelial adhesion, gastric and bile salt tolerance, pathogenicity, haemolytic activity, bacteriocin production, etc.) of the completed/draft genome.

The function can be verified through functional annotation to confirm the genetic arsenal of the strain required for gut adaptation [7,12,13]. Further functional gene prediction supports can serve as molecular markers in the screening and selection of probiotics [6,14]. *L. plantarum* represents one of the predominant genera with the largest microbial genome, of which 300 *L. plantarum* have completed the whole genome sequencing and are publicly available. To date, more than 100 *L. plantarum* genomes from different plant, animal and food sources from the Republic of Korea have been sequenced and analyzed [8,9,15]. Although *L. plantarum* finds wide application in Korean dairy products, to our surprise, no study has been carried out for genome sequencing and analysis of *L. plantarum* from raw milk. This, in fact, reflected the total 105 *L. plantarum* genomes available on the PATRIC database. To our knowledge, this is the first *L. plantarum* (designated as DJF10) from Korean raw milk subjected to whole genome sequencing and several bioinformatics analyses to gain an insight into the genomic features, specifically genes conferring probiotic- and safety-relevant properties.

## 2. Results and Discussion

### 2.1. Genome Characteristics of L. plantarum DJF10

**Figure 1:**
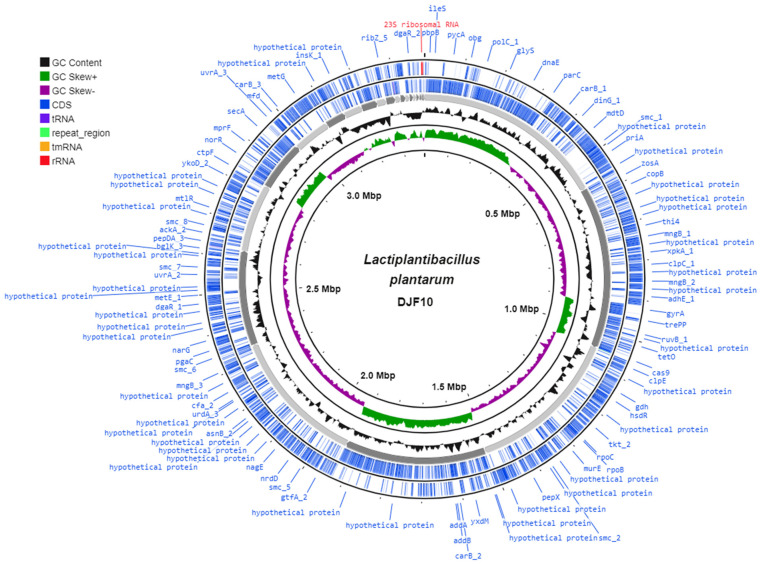
Circular map of the *Lactiplantibacillus plantarum* DJF10 genome visualized using the CGView tool. Genomic features marked from the outer to the inner circle as follows, circle 1 and 2 illustrate Prokka annotated forward and reverse CDS (coding sequences), respectively, with tRNA, rRNA, tmRNA and Cas elements; circle 3 represents GC content; circle 4 shows the GC skew (G-C)/(G+C), and circle 5 shows the genome size (3,385,113 bp).

The complete genome of *L. plantarum* DJF10 contains a single circular chromosome of 3,385,113 bp with a guanine-cytosine (GC) ratio of 44.3% (Figure 1). The complete genomic sequences of *L. plantarum* DJF10 have been submitted to NCBI SRA (Sequence Read Archive) submission portal under accession number SRR14598288. Our genome size was large in comparison to *L. plantarum* KLDS1.0391 [16], Y44 [17], DLK3 and JGR2 [18]. Earlier studies stated a positive connection between genome size/gene number and ubiquity, signifying that microbes with a large genome size can adapt well to various environments [16]. Interestingly, the GC content was in the same range as the foresaid *L. plantarum* stains. Microorganisms with a high GC content may consume more energy during reproduction, whereas in low GC content microbes, it is easier to maintain genomic stability due to low energy metabolism [19].

### 2.2. Species Confirmation

**Figure 2:**
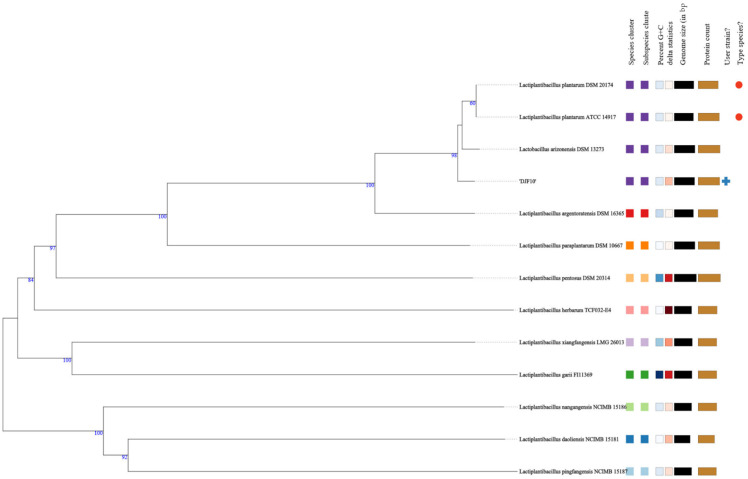
Phylogenetic comparison of *Lactiplantibacillus plantarum* DJF10 with representative complete genomes of other *Lactiplantibacillus* strains carried out in TYGS webserver. The tree was inferred with FASTME 2.1.6.1. from GBDP distances calculated from 16S rDNA gene sequences. The bootstrap support value before each node represents the confidence degree of each branch.

The genome similarity indices, ANI, were calculated between the submitted genome and genome sequences available in the JSpecies server [20]. The ANI values >95–96% were most often used as the criterion to confirm the species. DJF10 strain showed the highest ANI values for *Lactiplantibacillus plantarum* PS128 (ANIb 98.85%), *L. plantarum* 4_3 (ANIm 99.26%), and *L. plantarum* DSM 13273 (Tetra 0.99945). Further, phylogenomic analysis using genome-genome comparisons in TYGS revealed that the DJF10 strain is most closely related to *Lactiplantibacillus plantarum* DSM 20174 and ATCC 14917 strains (Figure 2). These results supported that the DJF10 strain unequivocally belongs to *L. plantarum*.

### 2.3. Annotation

Genome annotation using Prokka predicted a total of 3235 genes, including 3168 protein-coding sequences (CDS), 59 tRNAs, 7 rRNAs and 1 tmRNA (Table 1). No plasmid sequences were found in the genome. Of the predicted CDS, 1873 genes (59.12%) were functional, and 1295 genes (40.88%) were hypothetical/unknown. The 59 tRNA sequences correspond to 21 natural amino acids: Leu (6 sequences); Arg (5); Asn, Gly, Lys, Met, Ser, and Thr (4); Asp, Gln, Glu, Pro and Val (3); His, Phe, Tyr (2); Ala, Cys, Ile, Trp (1); and an undetermined protein (1).

Analysis of the genome on RAST provided an overview of the coded biological features with a subsystem coverage of 24%, distributed in 232 SEED subsystems (Table 2). The distribution of different functional groups showed a predominance of genes involved in general processes related to carbohydrates, amino acids and derivatives, and protein metabolism. Interestingly, 106 genes involved in the synthesis of cofactors, vitamins, prosthetic groups and pigments were unveiled. Notably, these genes were involved in the biosynthesis of biotin, thiamin, pyridoxine and folate, suggesting the ability of DJF10 to synthesize and transport B vitamins, a desirable trait of a probiotic strain.

#### 2.3.1. Functional Prediction

**Figure 3:**
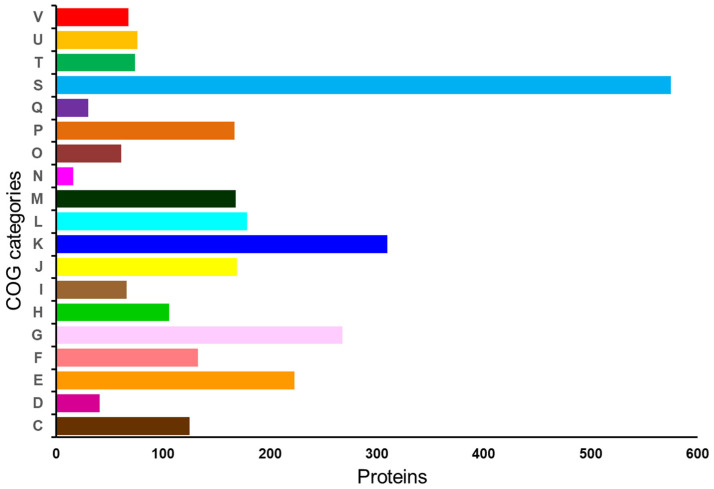
Distribution of Cluster of Orthologous group (COG) functional categories to the proteins of *Lactiplantibacillus plantarum* DJF10. In the y-axis, C denotes energy production and conversion, D—cell cycle control, cell division, and chromosome partitioning; E—amino acid transport and metabolism; F—nucleotide transport and metabolism; G—carbohydrate transport and metabolism; H—coenzyme transport and metabolism; I—lipid transport and metabolism; J-translation, ribosomal structure and biogenesis; K—transcription; L—replication, recombination and repair; cell wall/membrane/envelope biogenesis; N—Cell motility; O—posttranslational modification, protein turnover, chaperones; P—inorganic ion transport and metabolism; Q—secondary metabolites biosynthesis, transport and catabolism; S—function unknown; T—signal transduction mechanisms; U—intracellular trafficking, secretion, and vesicular transport; and V—defense mechanisms.

Analysis of the clusters of orthologous groups (COG) functional groups using EggNOG mapper v2 assigned the 2855 genes (90.12%) into 19 clusters (Figure 3). The higher numbers were sorted under Function unknown (S: 575), which confirms the uniqueness and unknown potential of this strain. Of note, a keen examination of this group revealed several loci of interest, including phage–related proteins, CRISPR-associated proteins, transport-related proteins and stress-related proteins. The remaining proteins were categorized under functional groups such as transcription (K: 310); amino acid transport and metabolism (E: 223); replication, recombination and repair (L: 179); translation, ribosomal structure and biogenesis (J: 169); cell wall/membrane/envelope biogenesis (M: 168); inorganic ion transport and metabolism (P: 167); carbohydrate transport and metabolism (G: 142); nucleotide transport and metabolism (F: 133); coenzyme transport and metabolism (H: 106); intracellular trafficking, secretion, and vesicular transport (U: 76); signal transduction mechanisms (T: 74); defense mechanisms (V: 68); lipid transport and metabolism (I: 66); posttranslational modification, protein turnover, chaperones (O: 61); energy production and conversion (C: 54); cell cycle control, cell division, chromosome partitioning (D: 41); secondary metabolites biosynthesis, transport and catabolism (Q: 30), and Cell motility (N: 12).

Furthermore, the KEGG functional annotation by BLASTKOALA assigned approximately half of the genes (52.1%, 1669 genes) into 22 different functional categories (Table 3), mostly related to protein families: genetic information processing (19.7%), carbohydrate metabolism (19.4%), protein families: signaling and cellular processes (15.8%), environmental information processing (14.1%), genetic information processing (13.8%), amino acid metabolism (8.3%), nucleotide metabolism (5.8%), among others.

The functional annotations by both COG and KEGG search indicate DJF10 has a high metabolic capacity, clarified by the high number of genes associated with genetic information and processing (J, K, L), followed by transport and metabolism (E, P, G, F, H) suggesting their importance in conserved cellular processes of our strain to take advantage in a large variety of niches.

#### 2.3.2. Probiotic Properties

The probiotic lactobacilli strains often harbor a huge number of genes encoding proteins involved in stress responses (temperature, pH, bile, osmotic pressure and oxidative stress) that regulate their adaptability to the GI tract. Based on published literature data, we searched the DJF10 genome for various probiotic property-related genes (stress resistance, bile salt hydrolase activity, adhesion ability, immunomodulatory activities) to determine its probiotic functions at genomic levels. We identified several genes encoding stress-related proteins in our genome, as listed in Table 4.

DJF10 was found to encode twelve genes related to heat shock proteins, including heat shock-related regulators (*hrcA*, *ctsR*), molecular chaperones (*dnaK*, *dnaJ*, *grpE*, *groEL*, *groES*, *hslO*) and protease-encoding genes (*hslU*, *hslV*, *lon*, *clpB*, *clpC*, *clpE*, *clpL*, *clpP* and *clpX*). These genes play a major role in intracellular protein aggregation and membrane stabilization to resist higher temperatures in *Lactobacillus* strains [21,22]. At the same time, DJF10 also contains five genes (*cspA*) coding for cold shock proteins related to survival under low temperatures. The CSP family genes are synthesized in several *L. plantarum* strains [16,23,24] to overcome the deleterious effect under cold stress, and hence, DJF10 may have the same function.

The DJF10 genome possessed ten genes encoding resistance at low pH conditions. Out of them, eight genes encode a cluster of F_0_F_1_ ATP syntheses subunit A–H, which serve as a key regulator of cytoplasmic pH to favor acid tolerance [25]. In addition, a *Nhac* gene coding for sodium-proton (Na^+^/H^+^) antiporters (maintain pH and Na^+^ homeostasis), an alkaline shock protein gene (Asp 23) and *gadB* gene coding glutamate decarboxylase were identified. All these genes confirm the DJF10 strain’s ability to adapt to an acidic environment. Regarding bile salt resistance, *cbh* coding cholylglycine hydrolase (converts conjugated bile acid into free bile acid), *ppaC* coding inorganic pyrophosphatase (maintain surface tension and keep membrane integrity), and *cfa* coding cyclopropane-fatty-acyl-phospholipid [CFP] synthase (enhance lipid synthesis), were identified. A similar bile-resistance mechanism was found in *L. petauri* LZys1 by genome and phenotype analysis [22]. Further, *opuA*, *opuC* and *opuBD* genes, known to shield against osmotic stress environments, were encoded in DJF10.

The ability of probiotic strains to adhere to the host epithelium is attributable to their cell surface proteins. DJF10 contains 13 genes putatively coding for adhesion-related proteins, including maltose phosphorylase (mapA), lipoprotein signal peptidase II (lspA), elongation factor Tu (tuf), L-glyceraldehyde 3-phosphate reductase (gpr), sortase A (srtA), LPXTG motif and putative glycosyltransferase (*EpsH*), providing evidence of high adhesion ability. In addition, a gene involved in gut persistence (*xylA*) was also present in the genome. Such adhesion-related genes were also reported in *L. plantarum* strains demonstrating good adhesion ability [25].

In general, *L. plantarum* suffers from unavoidable oxidative stress caused by excess accumulation of Reactive oxygen species (ROS) and Reactive nitrogen species (RNS) during cellular metabolism that could significantly damage DNA, proteins, and cell membranes [26]. *L. plantarum* DJF10 harbor 16 genes related to oxidative stress, and out of them, 8 encode the complete thioredoxin (*tpx*, *trxA*, *trxB*), glutathione (*gpx*, *gsr*), and NADH (*ndh*, *npr*, *nox*) antioxidant systems involved in ROS scavenging (Table 4). *Lactobacillus* sp., with a complete thioredoxin system, can remove both ROS and RNS at a higher reaction rate by donating electrons to thiol-dependent peroxidases. The glutathione system detoxifies hydrogen peroxide and lipid peroxyl radicals by regulating the protein dithiol/disulfide balance. The NADH oxidase/peroxidase and catalase can involve directly in hydrogen peroxide and ROS degradation [17,26]. In addition, DJF10 encoded genes for catalase (*katE*), pyruvate oxidase (*poxL*) and glutaredoxin (*nrdH*). Consistent with the previous observations in other *L. plantarum* strains [17,23,27], DJF10 lacks superoxide dismutase and compensates the enzyme with genes [manganese transport systems (*mntA-C*) and protein (*mntH*)] coding Mn^2+^ accumulation system. Interestingly, methionine sulfoxide reductase genes (*msrA*, *msrB*, *msrC*) that can repair the oxidized methionine residues by ROS in proteins were detected. The expression of these antioxidant genes was confirmed in the potential antioxidant *L. plantarum* Y44 [17] and STIII [25] genomes, which confer strain DJF10 ability with enhanced tolerance to oxidative stress. Moreover, the genes (*dlt* A-D) coding immunomodulatory activities were also identified in DJF10. These results suggest that *L. plantarum* DJF10 might resist multiple stress conditions and be consistent with the adaptability characteristics of the gastrointestinal tract. Further, the adhesion-related protein contributes to effective colonization of the intestinal environment and can devoid unwanted gut microorganisms.

### 2.4. Carbohydrate-Active Enzymes (CAZymes)

CAZymes are sequence-based classified enzymes that can synthesize, modify, and disintegrate complex carbohydrates and/or glycoconjugates, which widely exist in lactic acid bacteria. The analysis of the DJF10 genome in dbCAN2 webserver using the predicted amino acid sequences as input revealed a total of 98 genes classified under 5 different CAZymes gene families as follows: 54 glycoside hydrolase (GH) genes, 32 glycosyltransferase (GT) genes, 5 carbohydrate esterase (CE) genes, 4 carbohydrate-binding modules (CBMs), and 3 auxiliary activity (AA) genes. The large family in the genome was of GHs clustered in 27 families.

Further, a manual search revealed several transporter genes of beta-glucoside, cellobiose, fructose, galactitol, galactose, maltose and mannose, associated with phosphoenolpyruvate-dependent sugar phosphotransferase (PTS) system, the key carbohydrate active-transport system in bacteria. Additionally, several key enzymes associated with homo- and hetero-fermentation, such as acetate kinase, D and L-lactate dehydrogenases, glucose-6-phosphate isomerase, glucokinase, glyceraldehyde 3-phosphate dehydrogenase, phosphoglycerate kinase, phosphoketolase, pyruvate kinase and ribulose-phosphate 3-epimerase were identified, suggesting DJF10 can produce essential fermentation end-products in fermented food products.

### 2.5. Mobile Genetic Elements

#### 2.5.1. Insertion Sequences

A total of thirty-two insertion sequence (IS) elements belonging to six families (IS30, IS3, ISL3, IS256, IS1182, IS5) were predicted in the genome with the set threshold E-value of 0.00001 (Supplementary Table S1). The IS30 family was diverse, with three copies of each ISLpI1 and ISPp1, two copies of ISLsa1 and a single copy of ISLhe30 for was observed. Further, the genome contained fifteen copies of ISP2 in the family IS1182, two copies of each IS1310 and ISLPI2 element, and a single copy of the remaining five IS elements. Of the above-mentioned IS elements, ten insertion sequences exhibited a maximum score bit (>1000) with zero E-value. Similar IS elements have been frequently reported in other *L. plantarum* strains [14,18,28] that pose no safety risk.

#### 2.5.2. Island Viewer

Moreover, the profound analysis using Island viewer 4 did not detect virulence factors or pathogen-associated genes in the DJF10 genome. The identified genes were mapped into 18 different genomic islands, ranging in length from 4228 to 69,769 bp (Supplementary Table S2). The majority of CDSs were annotated as hypothetical proteins followed by antioxidant genes, bacteriocins, insertion sequences, stress-related proteins, sporulation proteins, enzymes related to carbohydrate metabolism, transporters, etc., aiding to increased adaptability of the organism in the environmental niche [29].

#### 2.5.3. CRISPR–Cas

The CRISPR-CasFinder identified three CRISPR arrays in the *L*. *plantarum* DJF10 genome, out of which only one located in contig 2 (start at 480,884, 36 bp repeat length, 12 repeats) matches a consensus sequence with evidence level 4 (Table 5). Additional CRISPR arrays detected in contig 5 were disregarded, as they are potentially invalid (evidence level 1). One mandatory CRISPR-associated protein of the CAS system (Cas2_TypeI-II-III), and three Cas-Type II systems that includes cas1, cas2, cas9, and csn2 were predicted within contig 2 (Table 5). The detected Crispr-Cas systems in the DJF10 strain implicate the ability to protect themselves against foreign genetic elements (phages, plasmids and insertion sequences), along with prevention of the strain from acquiring resistance genes (virulence, antibiotic) through horizontal gene transfer [15].

#### 2.5.4. Prophages

Screening for prophage sequences in the DJF10 genome using PHASTER revealed three regions (Table 6), including two intact (regions 1 and 3) and one questionable (region 2). Each phage region is mainly composed of bacteriophage proteins and hypothetical proteins from diverse genera, including *Lactobacillus*, *Enterococcus*, and *Bacillus*. The highest (53.9 kb) intact phage in region 3 (155,716–209,682 bp) showed a maximum (56) protein matching and resembled Lactob_Sha1_NC_019489(32), the most encountered prophage in several *L. plantarum* strains [30]. The next intact phage at region 1 (377,986–394,877 bp, 31 proteins; 16.8 kb) resembled PHAGE_Entero_phiSHEF4_NC_042022(2). The questionable phage in region 2 (262,426–282,208 bp, 22 proteins; 19.7 kb) corresponded to PHAGE_Entero_vB_EfaS_AL2_NC_042127(2). All the 3 prophages in DJF10 genome showed the presence of an integrase [PP_00982 (region 1), PP_01833 (region 2) and PP_02616 (region 3)]. In bacterial genomes, integrases act as functional markers for prophages, pathogenicity islands and integrative plasmids [23,31]. The phage attachment sites (attL/attR) in all three regions were located upstream of the integrase. In addition, attL and attR sequences were identical in each intact phage. No virulence or AMR genes, have been found within the intact prophages.

### 2.6. Safety-Associated Genes

#### 2.6.1. Antimicrobial Resistance (AMR) Genes

No AMR genes were found in Resfinder 4.1 database (90% threshold and 60% minimum length). While only one gene coding *vanY,* was detected in the CARD database search under default parameters (perfect and strict hits, only). However, changing the parameter to perfect, strict and loose hits resulted in 198 hits (1 strict + 197 loose hits), with an identity of the matching region ranging from 20–71% and coverage of 24–318%. The loose hits (Supplementary Table S3) included genes of resistance mechanism to antibiotic target alteration (40), antibiotic target protection (12), antibiotic efflux (134), reduced permeability to antibiotic (1), antibiotic inactivation (7) and antibiotic target replacement (4). Since both the above databases mainly focus on the AMR genes of pathogenic bacteria, the AMR genes of non-pathogenic bacteria (e.g., *Lactobacillus*) are usually not included.

In this regard, the KEGG database search yielded fifteen AMR-related genes in the DJF-10 genome (Table 7). The identified genes were related to vancomycin (*vanY*, *vanX*), chloramphenicol (*catA*), tetracycline (*tetM*, *tetO*) and beta-lactam (*penP*) resistance. Vancomycin resistance is intrinsic in *L. plantarum* strains that produce D-lactate-ended peptidoglycan precursors, instead of D-alanine at the C-terminus [32]. The *vanY* and *vanX* genes coded for D-Ala-D-Ala carboxypeptidase and D-Ala-D-Ala dipeptidase, respectively. Campedelli et al. [33] observed the *catA* gene in several lactobacilli strains, although they were susceptible to chloramphenicol. Furthermore, macrolide, multidrug and cationic antimicrobial peptide resistance genes reside in the genome. Chokesajjawatee et al. [34] stated that the macrolide and beta-lactamase resistance genes in the *L. plantarum* BCC 9546 genome did not guarantee the resistance for erythromycin and ampicillin, respectively. The authors explained that it could be related to varied factors such as gene expression level and substrate specificity of the expressed product. The phenotypic susceptibility of LAB strains to common antibiotics had been evidenced in a few earlier studies despite the occurrence of AMR genes, as the phenotype and genotype do not overlap completely. Hence, further investigations are needed to clarify whether these putative AMR genes encode active proteins or play different roles.

#### 2.6.2. Virulence Factors

No virulence genes were detected under the BLASTn search on the VirulenceFinder. A total of 16 virulence genes were predicted by VFDB, mainly associated with adherence, stress survival, iron uptake, and immune modulation. These genes were characterized as virulence factors in pathogens for survival in the host environment under physiological stresses; however, the same can favor a probiotic for survival in the gut [35].

#### 2.6.3. Toxins, Biogenic Amines and Undesirable Genes

The genes coding undesirable properties were identified using BlastKoala, which included hemolysins (tlyC—K03699; hlyIII—K11068), lactate racemase (K22373), D-lactate dehydrogenase (K03778) and choloylglycine hydrolase (K01442). Both hemolysins are widespread in several commercially availed probiotic *Lactobacillus* strains [36,37], implying their presence is not a safety concern. However, further verification is mandatory to confirm the hemolytic activity. The lactate racemase and D-lactate dehydrogenase genes code D-lactic acid production, which is an essential component of cell wall peptidoglycan in several Gram–positive bacteria, including *L. plantarum*. Since D-lactic production is an intrinsic property, precautions overconsumption of such LAB-included foods are mandatory for patients with a high risk of D-lactic acidosis [38]. BA production is another essential probiotic attribute related to safety issues. In the DJF10 genome, no genes related to BA production were detected, indicating the strain is a non-producer of BA and poses no safety threat in this aspect.

Furthermore, no plasmid was detected in the genome using the PlasmidFinder web tool [39], and the probability of being a human pathogen assessed using Pathogen Finder [40] was near zero (0.207) indicating the safe use of DJF10 in food and beverage applications.

### 2.7. Bacteriocin-Encoding Genes

**Figure 4:**
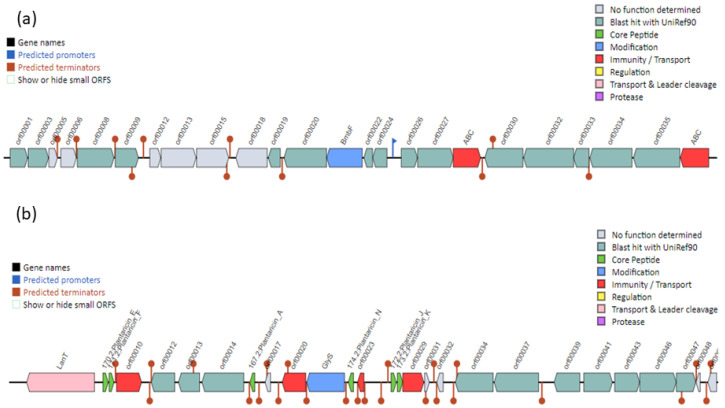
The organization of bacteriocin gene clusters in the *Lactiplantibacillus plantarum* DJF10 genome was predicted through the BAGEL4 webserver. The area of interest at contig 6 and 10 indicates sacpeptides (**a**) and plantaricin J (**b**) classes, respectively.

The blast results of the BAGEL4 webserver for *L. plantarum* DJF10 genome predicted two bacteriocin clusters as Areas of interest (AOI’s) at (i) contig 6.17 (start at 53,360 and end at 73,360) (ii) contig 10.2 (start at 0 and end 22,468) (Figure 4). The contig 6.17 encodes bacteriocin of the sactipeptides class (ribosomally synthesized and post-translationally modified peptides), which resides a BmbF gene, two ABC transporter ATP-binding proteins, and several ORFs (Open Reading Frames) (Figure 4a). This structure seems similar to the sactipeptides from *L. plantarum* UTNGt2 [41].

The second AOI located at contig 10.2 encodes the bacteriocin of Plantaricin J class that resides in several core peptides (*pln EF*, *pln A*, *pln N*, and *pln JK*) (Figure 4b). Interestingly, the same core peptides have been reported earlier in the genome of *L. plantarum* FLPL05, WSFS1 and ATCC14917, although their gene orientation was a reversal to DJF10. The two-peptides bacteriocins, *pln EF* and *pln JK* belong to class IIb bacteriocin, while *pln A* and *pln N* to class IId, implying that *L. plantarum* DJF10 might be a producer of bacteriocins, especially for class II bacteriocins. These peptides inhibit both Gram-positive and Gram-negative bacteria [25]. The isoelectric point (pI) and length of the foresaid core genes (amino acids) without GG leader sequence detected in our study (Supplemental Table S4) are identical to the earlier report [42].

The protein sequences of all members in the gene cluster were confirmed by Blastp (Supplemental Table S5). In addition to the aforesaid core peptides, the bacteriocin structure included four immunity proteins (*pln I*, *pln P*, *pln M*, and *pln L*), two response regulators (*pln D* and *pln C*), a modified peptide of glycosyltransferase family 2 protein (GlyS), and a transporter (*pln G*) LanT, Bacteriocin ABC-transporter, ATP-binding and permease protein. Further, ORFs encoding for plantaricin biosynthesis proteins (*pln Q* and *pln R*), putative Na^+^/H^+^ antiporter protein, histidine protein kinase, MFS transporter, sugar O-acetyltransferase, and two Cof-type HAD-IIB family hydrolases were also present. The other members of the bacteriocin gene cluster are given in Supplemental Table S5.

## 3. Materials and Methods

### 3.1. Strain Information

The strain was isolated from raw milk obtained in the dairy farms of the Republic of Korea. For isolation, the samples were serially diluted and plated on lactobacilli MRS agar (Difco, Franklin Lakes, NJ, USA) at 37 °C for 48 h under anaerobic conditions. The morphologically different colonies were selected and restreaked on MRS agar plates to obtain a pure, single colony. The isolates were screened for their ability to survive under 2 mM H_2_O_2_, and the selected isolate (designated as DJF10) was stored at −80 °C until further studies.

### 3.2. DNA Extraction, Whole Genome Sequencing, Assembly and Annotation

Genomic DNA was isolated from the DJF10 strain (grown in MRS broth aerobically at 37 °C, 16 h) using Exgene^TM^ Cell SV Kit (Cambio, Reading, UK) according to the manufacturer’s protocol. The DNA extract was quantified using the Quant-iT^TM^ BR assay Kit (Invitrogen, Waltham, MA, USA). A standard genomic Illumina 350 bp paired-end library was constructed from the chromosomal DNA and sequenced using Illumina Novaseq 6000 platform at TK Biotech and science (Jeonbuk, Republic of Korea), and the raw reads were uploaded in Galaxy (usegalaxy.org; accessed on 17 August 2022) and using the Trimmomatic tool v0.38.1 (http://www.usadellab.org/cms/?page=trimmomatic, accessed on 17 August 2022), data were filtered for adapter sequences, and low-quality reads. Then de novo assembly was performed with Shovill v1.1.0 [43] under default parameters by excluding contigs shorter than 100 bp. The quality of the assembled sequence was assessed using the Quast v5.2.0 tool. A circular genomic map was constructed from the resultant genome using the CG view server [44].

### 3.3. Genome-Based Identification

To identify the species, the average nucleotide identity (ANI) and tetra indices of the DJF10 strain were calculated in the JSpecies Web Server [20]. The Type (Strain) Genome Server (TYGS) [45] was also used to create a bootstrapped phylogenetic relationship through pairwise comparison of genome sequences.

### 3.4. Annotation and Functional Prediction

Genome annotation was carried out using the Prokaryotic Genome Annotation System (Prokka) v1.14.6 in Galaxy [46] and the Rapid Annotations using Subsystems Technology (RAST) webserver [47]. In addition, the functional annotations were carried out using the egg-NOG mapper [48] and the KEGG database [49]. Identification of the most important genes related to probiotic properties was manually predicted from Prokka, RAST and KEGG–derived annotations. Carbohydrate-active enzymes (CAZymes) within the DJF10 genome were identified using the CAZy database [50].

### 3.5. Genome Instability

The insertion elements in the genome were identified with the ISfinder databaseusing BLASTn v2.2.31 with an E-value threshold of 1 × 10^−5^ [51]. The Island viewer 4 server was employed to determine the genomic islands and the presence of genes related to pathogenicity [52]. Coding sequences for Clustered Regularly Interspaced Short Palindromic Repeats (CRISPR) and CRISPR-associated genes (Cas). were determined with an online detection tool, CRISPRCasFinder using default parameters [53]. Identification and annotation of prophage DNA sequences within the bacterial genome were achieved using the PHASTER (PHAge Search Tool Enhanced Release) [54].

### 3.6. Safety Assessment

The search for antimicrobial resistance (AMR) genes in the DJF10 genome was carried out in three publicly available databases, i.e., ResFinder tool v.4.1. of the Center for Genomic Epidemiology [55], Resistance Gene Identifier (RGI) tool in the Comprehensive Antibiotic Resistance Database (CARD) [56] and BlastKOALA tool in the KEGG database [49].

Putative virulence factors were determined using the web tools of Virulence finder v.2.0.3 [57] and the Virulence Factor of Bacterial Pathogen database (VFDB) [58].

The search for genes involved in toxins, biogenic amine (BA) production and other undesirable properties were done using the BlastKOALA tool in the Kyoto Encyclopedia of Genes and Genomes (KEGG) database [49] as stated in Chokesajjawatee et al. [34].

### 3.7. Secondary Metabolites

The prediction of bacteriocin-related genes in the genome of DJF10 was fulfilled using the BAGEL 4 webserver (http://bagel4.molgenrug.nl/, accessed on 19 August 2022) [59]. Subsequently, each ORF in the predicted bacteriocin structure was verified via BLASTp (https://blast.ncbi.nlm.nih.gov/Blast.cgi, accessed on 19 August 2022). The molecular mass (MW) and theoretical isoelectric point (pI) of predicted peptides were computed in the Expasy Compute pI/MW online tool (https://www.expasy.org/resources/compute-pI-mw, accessed on 19 August 2022).

## 4. Conclusions

Overall, the study reports on isolation, whole genome sequencing, and bioinformatics analysis of the *L. plantarum* DJF10 strain. To the best of our knowledge, this is the first genomic study of *L. plantarum* isolated from Korean raw milk. The assembled genome consists of twenty-nine contigs, and one chromosome, with a total length of 3,385,113 bp and an average G+C content of 44.3%. Annotation of the assembled genome using Prokka and RAST; and further functional annotation by EggNOG mapper and BLASTKOALA provided a comprehensive perspective on the lifestyle of DJF10. The genome presents a variety of genes associated with probiotic survival (acid, bile, temperature, oxidative stress, bacteriocin production) and adaption (adhesion and carbohydrate-active enzymes) properties indicating DJF10 as a potential probiotic candidate. At the same time, prophage regions, insertion sequences, and CRISPR-Cas systems related to genome stability were revealed. Relating to safety concerns, the plasmids and genes related to virulence factors, toxins, and biogenic amine production were absent. Although DJF10 confirms as a non-human pathogen, hemolysin and antibiotic-resistance genes determined in the KEGG search need further experimental validation. The outcome of this study significantly improved the knowledge of the genetic characteristics of this promising strain. Nonetheless, it is necessary to further elucidate the potential health benefit and application as a probiotic strain in food industries, through in vitro and in vivo experiments.

## Figures and Tables

**Table 1:** General genome features of *Lactiplantibacillus plantarum* DJF10.

Attribute	Value
Genome size (bp)	3,385,113
GC content (%)	44.3
Number of contigs	29
N50 (bp)	418,773
L50	4
Plasmids	0
CDS	3168
Total RNA’s	68 (60 tRNA + 7 rRNA + 1 tmRNA)
Total genes	3235
Protein-coding genes	3168
Genes assigned to COGs	2855

**Table 2:** General overview of biological subsystem distribution of the genes by RAST annotation.

Description	Value	Percent
Cofactors, Vitamins, Prosthetic Groups, Pigments	106	9.5
Cell Wall and Capsule	52	4.6
Virulence, Disease and Defense	41	3.7
Potassium metabolism	6	0.5
Miscellaneous	14	1.3
Phages, Prophages, Transposable elements, Plasmids	9	0.8
Membrane Transport	35	3.1
Iron acquisition and metabolism	5	0.4
RNA Metabolism	38	3.4
Nucleosides and Nucleotides	91	8.1
Protein Metabolism	127	11.3
Cell Division and Cell Cycle	4	0.4
Regulation and Cell signaling	16	1.4
Secondary Metabolism	4	0.4
DNA Metabolism	63	5.6
Fatty Acids, Lipids, and Isoprenoids	34	3.0
Nitrogen Metabolism	8	0.7
Dormancy and Sporulation	6	0.5
Respiration	16	1.4
Stress Response	20	1.8
Metabolism of Aromatic Compounds	8	0.7
Amino Acids and Derivatives	175	15.6
Sulfur Metabolism	4	0.4
Phosphorus Metabolism	7	0.6
Carbohydrates	230	20.55

**Table 3:** KEGG orthology (KO) categories of identified protein-coding genes in the *Lactiplantibacillus plantarum* DJF10 genome.

KO Number	Functional Category	Gene Number	Proportion (%)
09101	Carbohydrate metabolism	226	13.59
09102	Energy metabolism	37	2.22
09103	Lipid metabolism	39	2.35
09104	Nucleotide metabolism	68	4.09
09105	Amino acid metabolism	97	5.83
09106	Metabolism of other amino acids	20	1.20
09107	Glycan biosynthesis and metabolism	37	2.22
09108	Metabolism of cofactors and vitamins	65	3.91
09109	Metabolism of terpenoids and polyketides	10	0.60
09110	Biosynthesis of secondary metabolites	5	0.30
09111	Xenobiotics biodegradation and metabolism	8	0.48
09120	Genetic information processing	161	9.68
09130	Environmental information processing	164	9.86
09140	Cellular processes	11	0.66
09150	Organismal systems	8	0.48
09160	Human diseases	3	0.18
09181	Protein families: metabolism	39	2.35
09182	Protein families: genetic information processing	229	13.77
09183	Protein families: signaling and cellular processes	184	11.06
09191	Unclassified: metabolism	110	6.61

**Table 4:** List of probiotic marker genes identified in the *Lactiplantibacillus plantarum* DJF10 genome.

Gene	Function	Gene Nos.
Temperature		
*Heat stress*		
*htpX*	heat shock protein HtpX	1
*hrcA*	heat-inducible transcriptional repressor	1
*hslO*	molecular chaperone Hsp33	1
*hslU*	ATP-dependent HslUV protease ATP-binding subunit HslU	1
*HSP20*	HSP20 family protein	3
*dnaK*	HSPA9; molecular chaperone DnaK	1
*dnaJ*	molecular chaperone DnaJ	1
*ctsR*	transcriptional regulator of stress and heat shock response	1
*grpE*	molecular chaperone GrpE	1
*groEL*	HSPD1; chaperonin GroEL	1
*groES*	HSPE1; chaperonin GroES	1
*lon*	Lon-like protease	1
*clpB*	ATP-dependent Clp protease ATP-binding subunit ClpB	1
*clpC*	ATP-dependent Clp protease ATP-binding subunit ClpC	1
*clpE*	ATP-dependent Clp protease ATP-binding subunit ClpE	1
*clpL*	ATP-dependent Clp protease ATP-binding subunit ClpL	1
*clpX*	ATP-dependent Clp protease ATP-binding subunit ClpX	1
*clpP*	ATP-dependent Clp protease, protease subunit	1
*hslV*	ATP-dependent HslUV protease, peptidase subunit HslV	1
Cold stress		
*cspA*	cold shock protein	5
Acid stress		
*atpA*	F-type H^+^/Na^+^-transporting ATPase subunit alpha	1
*atpB*	F-type H^+^-transporting ATPase subunit a	1
*atpE*	F-type H^+^-transporting ATPase subunit c	2
*atpF*	F-type H^+^-transporting ATPase subunit b	1
*atpH*	F-type H^+^-transporting ATPase subunit delta	1
*atpG*	F-type H^+^-transporting ATPase subunit gamma	1
*atpD*	F-type H^+^/Na^+^-transporting ATPase subunit beta	1
*atpC*	F-type H^+^-transporting ATPase subunit epsilon	1
*gadB*	gadB, gadA, GAD; glutamate decarboxylase	1
*nhaC*	Na^+^:H^+^ antiporter, NhaC family	1
Bile tolerance		
*cbh*	choloylglycine hydrolase	1
*ppaC*	manganese-dependent inorganic pyrophosphatase	1
*cfa*	cyclopropane-fatty-acyl-phospholipid synthase	1
Adhesion		
*mapA*	maltose phosphorylase	2
*lspA*	lipoprotein signal peptidase II	1
*tuf*	elongation factor Tu	1
*gpr*	L-glyceraldehyde 3-phosphate reductase	1
*tpiA*	triosephosphate isomerase (TIM)	1
*gapA*	glyceraldehyde 3-phosphate dehydrogenase (phosphorylating)	1
*bgaB*	beta-galactosidase	1
*srtA*	sortase A	1
*epsA*	protein tyrosine kinase modulator	2
*epsB*	protein-tyrosine kinase	2
*pgaC*	poly-beta-1,6-N-acetyl-D-glucosamine synthase	3
*eno*	enolase	2
*pgi*	glucose-6-phosphate isomerase	1
*LPXTG*	surface protein (LPXTG motif)	1
epsH	Putative glycosyltransferase EpsH	1
Antioxidant		
*katE*	catalase	1
*fnr*	ferredoxin/flavodoxin—NADP^+^ reductase	5
*nrdH*	glutaredoxin	2
*gpx*	glutathione peroxidase	1
*gsr*	glutathione reductase	2
*mntH*	manganese transport protein	2
*mntA*	manganese transport system ATP-binding protein	1
*mntB*	manganese transport system permease protein	5
*mntC*	manganese transport system substrate-binding protein	2
*ndh*	NADH dehydrogenase	1
*npr*	NADH peroxidase	2
*poxL*	pyruvate oxidase	1
*tpx*	thiol peroxidase	1
*trxA*	thioredoxin	1
*trxB*	thioredoxin reductase	3
*nox*	nadh oxidase	4
*msrA*	peptide-methionine (S)-S-oxide reductase	2
*msrB*	peptide-methionine (R)-S-oxide reductase	1
*msrC*	L-methionine (R)-S-oxide reductase	1
Immunomodulation		
*dltA*	D-alanine—poly(phosphoribitol) ligase subunit 1	1
*dltB*	membrane protein involved in D-alanine export	1
*dltC*	D-alanine—poly(phosphoribitol) ligase subunit 2	1
*dltD*	D-alanine transfer protein	1

**Table 5:** Crispr array system (a) and Cas type detection (b) within *Lactiplantibacillus plantarum* DJF10 genome using CRISPRFinder tool.

(a)									
CRISPR_ID	Start	End	Length (bp)	Orientation	Consensus Repeat	No. of CRISPRs with Same Repeat (Crisprdb)	Repeat Length	No. of Spacers	Evidence Level
Contig 2_1	480,884	481,711	827	Forward	GTCTTGAATAGTAGTCATATCAAACAGGTTTAGAAC	4	36	12	4
Contig 5_1	73,501	73,616	115	Forward	CTTGAACCAGCAAAGAGTTGTTGAACTGCACT	0	32	1	1
Contig 5_2	75,535	75,647	112	Unknown	GAACCATCAGCCAACTGACTGACACCACT	0	29	1	1
**(b)**									
**Sequence ID**	**Cas-Type/Subtype**			**Gene Status**	**System**	**Type**	**Begin**	**End**	**Strand**
Contig 2_434	cas2_TypeI-II-III			mandatory	CAS	CDS	479,880	480,185	+
Contig 2_433	cas1_TypeII			forbidden	CAS-TypeIIU	CDS	478,987	479,823	+
Contig 2_432	cas9_TypeII			forbidden	CAS-TypeIIU	CDS	474,716	478,792	+
Contig 2_435	csn2_TypeIIA			forbidden	CAS-TypeIIA	CDS	480,182	480,859	+

**Table 6:** Prophage regions of *Lactiplantibacillus plantarum* DJF10 identified in PHASTER.

Region	Region Length (kb)	Completeness	Score	Total Proteins	Region Position (bp)	Most Common Phage (Number of Matching Proteins)	GC%	attL/attR Sites	Integrase ORF Start–Stop
1	16.8	intact	100	31	377,986–394,877	PHAGE_Entero_phiSHEF4_NC_042022(2)	41.69	+	379,616–380,770
2	19.7	Questionable	70	22	262,426–282,208	PHAGE_Entero_vB_EfaS_AL2_NC_042127(2)	41.62	+	262,710–263,867
3	53.9	intact	150	56	155,716–209,682	PHAGE_Lactob_Sha1_NC_019489(32)	42.26	+	205,632–206,768

**Table 7:** AMR (antimicrobial resistance) genes identified and their location in the *Lactiplantibacillus plantarum* DJF10 genome.

Resistance	Description	KEGG_ID	Gene Name	Gene Location
Tetracycline resistance	Others	K18220	tetM, tetO; ribosomal protection tetracycline resistance protein	contig00002_393
Macrolide resistance	Transporters	K18231	msr, vmlR; macrolide transport system ATP-binding/permease protein	contig00007_107
		K08217	mef; MFS transporter, DHA3 family, macrolide efflux protein	contig00004_31
Phenicol resistance	Acetyltransferases	K19271	catA; chloramphenicol O-acetyltransferase type A [EC:2.3.1.28]	contig00001_343
beta-Lactam resistance	Bla system [MD:M00627]	K17836	penP; beta-lactamase class A [EC:3.5.2.6]	contig00004_310; contig00009_56
Vancomycin resistance	D-Ala-D-Lac type [MD:M00651]	K07260	vanY; zinc D-Ala-D-Ala carboxypeptidase [EC:3.4.17.14]	contig00003_219
		K08641	vanX; zinc D-Ala-D-Ala dipeptidase [EC:3.4.13.22]	contig00008_133
Cationic antimicrobial peptide (CAMP) resistance	dltABCD operon [MD:M00725]	K03367	dltA; D-alanine―poly(phosphoribitol) ligase subunit 1 [EC:6.1.1.13]	contig00001_166
		K03739	dltB; membrane protein involved in D-alanine export	contig00001_167
		K14188	dltC; D-alanine―poly(phosphoribitol) ligase subunit 2 [EC:6.1.1.13]	contig00001_168; contig00006_124
		K03740	dltD; D-alanine transfer protein	contig00001_169
	lysyl-phosphatidylglycerol (L-PG) synthase MprF [MD:M00726]	K14205	mprF, fmtC; phosphatidylglycerol lysyltransferase [EC:2.3.2.3]	contig00008_63
Multidrug resistance	efflux pump AbcA [MD:M00700]	K18907	norG; GntR family transcriptional regulator, regulator for abcA and norABC	contig00006_123
		K18104	abcA, bmrA; ATP-binding cassette, subfamily B, bacterial AbcA/BmrA [EC:7.6.2.2]	contig00004_233; contig00005_131
	efflux pump NorB [MD:M00702]	K18907	norG; GntR family transcriptional regulator, regulator for abcA and norABC	contig00006_123

## Data Availability

The complete genomic sequences of *L. plantarum* DJF10 have been submitted in NCBI SRA (Sequence Read Archive) submission portal under accession number SRR14598288; BioSample SAMN19277818 in BioProject PRJNA731289 on 20 May 2021.

## Supplementary Materials

The following supporting information can be downloaded at: https://www.mdpi.com/article/10.3390/ijms232214494/s1, Table S1: Insertion sequences found in the *Lactiplantibacillus plantarum* DJF10 genome using IS finder; Table S2: Genomic Islands found in the *Lactiplantibacillus plantarum* DJF10 genome using Island viewer; Table S3: AMR (loose hits) found in the *Lactiplantibacillus plantarum* DJF10 genome using CARD database; Table S4: The aminoacid sequences of the predicted bacteriocin and properties of the mature peptide sequences; Table S5: The protein- BLAST results for the members in the Plantaricin gene cluster of *Lactiplantibacillus plantarum* DJF10 predicted with BAGEL4 webserver.
