# Supplementary material for: Probiogenomic In-Silico Analysis and Safety Assessment of Lactiplantibacillus plantarum DJF10 Strain Isolated from Korean Raw Milk
Source: Int J Mol Sci. 2022 Nov 21;23(22):14494. doi: 10.3390/ijms232214494 (PMC9699202; doi:10.3390/ijms232214494)
Supplement: Supplementary file 1 [file ijms-23-14494-s001.zip › Table S4.pdf]

Table S4. The aminoacid sequences of the predicted bacteriocin and properties of the mature peptide sequences

Bacteriocin	Aminoacid sequence*	Length of the mature product (aminoacids)	pI of mature product	MW of the mature product (Da)
Plantaricin E	MLQFEKLQYSRLPQKKLAKIS**GG**FNRGGYNFGKSVRHVVDAIGSVAGIRGILKSIR	33	11.57	3545.11
Plantaricin F	MKKFLVLRDRELNAIS**GG**VFHAYSARGVRNNYKSAVGPADWVISAVRGFIHG	34	10.27	3703.18
Plantaricin A	VIIMKIQIKGMKQLSNKEMQKIV**GG**KSSAYSLQMGATAIKQVKKLFKKWGW	26	10.4	2985.58
Plantaricin N	MKSLDKIAGLGIEMAEKDLTTVE**GG**KNYSKTWWYKSLTLLGKVAEGTSSAWHGLG	30	9.7	3369.83
Plantaricin J	MTVNKMIKDLDVVDAFAPISNNKLNGVV**GG**GAWKNFWSSLRKGFYDGEAGRAIRR	25	10.93	2872.24
Plantaricin K	MKIKLTVLNEFEELTADAEKNIS**GG**RRSRKNGIGYAIGYAFGAVERAVLGGSRDYNK	32	10.52	3502.94

* The mature peptides without GG leader sequences underlined
