# Supplementary material for: Probiogenomic In-Silico Analysis and Safety Assessment of Lactiplantibacillus plantarum DJF10 Strain Isolated from Korean Raw Milk
Source: Int J Mol Sci. 2022 Nov 21;23(22):14494. doi: 10.3390/ijms232214494 (PMC9699202; doi:10.3390/ijms232214494)
Supplement: Supplementary file 1 [file ijms-23-14494-s001.zip › Table S5.pdf]

Table S5. The protein- BLAST results for the members in the Plantaricin gene cluster of *Lactiplantibacillus plantarum* DJF10 predicted with BAGEL4 webserver

#	Name	Gene start (bp)	Gene end (bp)	Gene strand	Function	E-value	Percent identity	Accession
1	orf00001 (LanT)	554	2704	-	Bacteriocin ABC-transporter, ATP-binding and permease protein PlnG [*Lactiplantibacillus plantarum*]	0	99.9%	MCG0835499.1
2	orf00003 (**170.2;Plantaricin_E)**	2971	3141	+	two-peptide bacteriocin plantaricin EF subunit PlnE [*Lactiplantibacillus*]	5E-31	100%	WP_003641985.1
3	orf00005 (**171.2;Plantaricin_F)**	3166	3324	+	two-peptide bacteriocin plantaricin EF subunit PlnF [*Lactiplantibacillus*]	5E-29	100%	WP_003643811.1
4	orf00010 (Plnl)	3405	4196	+	CPBP family glutamic-type intramembrane protease [*Lactiplantibacillus paraplantarum*]	4E-151	99.24%	WP_251898977.1
5	orf00012	4502	5245	-	response regulator PlnD [*Lactiplantibacillus plantarum*]	3E-178	99.19%	ADE08254.1
6	orf00013	5364	6032	-	response regulator PlnC, activator [*Lactiplantibacillus plantarum*]	2E-149	99.55%	MCG0835494.1
7	orf00014	6108	7436	-	histidine protein kinase [*Lactiplantibacillus plantarum*]	0	99.55%	ACO06046.1
8	orf00016 (**167.2;Plantaricin_A)**	7627	7782	-	hypothetical protein FC76_GL001049 [*Lactiplantibacillus plantarum* subsp. *plantarum* ATCC 14917 = JCM 1149 = CGMCC 1.2437]	1E-16	100%	KRL33425.1
9	orf00017	8109	8282	-	plantaricin biosynthesis protein PlnQ [*Lactiplantibacillus plantarum*]	3E-31	98.25%	ABD15220.1
10	orf00020 (PlnP)	8651	9397	-	CPBP family intramembrane metalloprotease [*Lactiplantibacillus plantarum*]	1E-132	98.79%	WP_047672663.1
11	orf00021 (GlyS)	9428	10627	-	glycosyltransferase family 2 protein [*Lactiplantibacillus plantarum]*	0	99.75%	WP_063204020.1
12	orf00022 (**174.2;Plantaricin_N**	10745	10912	-	bacteriocin [*Lactiplantibacillus*]	2E-29	100%	WP_003641975.1
13	orf00023	11040	11240	-	Immunity protein PlnM [*Lactiplantibacillus plantarum*]	1E-19	93.18%	KEZ15850.1
14	orf00027 (**172.2;Plantaricin_J)**	12102	12269	+	two-peptide bacteriocin plantaricin JK subunit PlnJ [*Lactiplantibacillus plantarum*]	7E-31	98.18%	WP_015379769.1
15	orf00028 (**173.2;Plantaricin_K)**	12300	12473	+	two-peptide bacteriocin plantaricin JK subunit PlnK [*Lactiplantibacillus plantarum*]	8E-20	98.25%	WP_196500391.1
16	orf00029 (PlnL)	12470	13138	+	bacteriocin immunity protein [*Lactiplantibacillus plantarum*]	2E-137	99.55%	WP_072540368.1
17	orf00031	13163	13315	+	plantaricin biosynthesis protein PlnR [*Lactiplantibacillus plantarum*]	1E-25	100 %	AAS21880.1
18	orf00032	13550	13753	-	hypothetical protein [*Lactiplantibacillus plantarum*]	3E-41	98.51%	WP_107787099.1
19	orf00034	14138	15334	-	putative Na(+)/H(+) antiporter [*Lactiplantibacillus plantarum* subsp. *plantarum*]	0	100%	AFM80194.1
20	orf00037	15367	16776	-	branched-chain amino acid transport system II carrier protein [*Lactiplantibacillus plantarum* subsp. *plantarum*	0	100%	EFK30741.1
21	orf00039	17269	18084	-	Cof-type HAD-IIB family hydrolase [*Lactiplantibacillus plantarum*]	0	99.61%	WP_162045214.1
22	orf00041	18208	19116	+	AraC family transcriptional regulator [*Lactiplantibacillus*	0	99.66%	APP12939.1

					plantarum subsp. *plantarum*]			
23	orf00043	19187	19978	+	Cof-type HAD-IIB family hydrolase [*Lactiplantibacillus plantarum*]	3E-166	100%	WP_072540366.1
24	orf00046	19982	21136	+	MFS transporter [*Lactiplantibacillus plantarum*]	0	99.74%	WP_063204022.1
25	orf00047	21140	21757	+	sugar O-acetyltransferase [*Lactiplantibacillus plantarum*]	3E-138	99.51%	WP_133279594.1
26	orf00048	21763	21900	-	hypothetical protein I526_0335 [*Lactiplantibacillus plantarum* DOMLa]	3E-23	97.78%	AHN68020.1
27	orf00052	22139	22417	-	hypothetical protein [*Lactiplantibacillus plantarum*]	6E-54	100%	WP_072540096.1
